# First identification of canine adenovirus 1 in mink and bioinformatics analysis of its 100 K protein

**DOI:** 10.3389/fmicb.2023.1245581

**Published:** 2023-08-17

**Authors:** Jinyu Hou, Jinfeng Xu, Ben Wang, Hongling Zhang, Baishuang Yin, Goujiang Li, Fashou Lei, Xiaoming Cai, Yanzhu Zhu, Longtao Wang

**Affiliations:** ^1^College of Veterinary Medicine, Jilin Agricultural University, Changchun, China; ^2^Animal Science and Technology College, Jilin Agriculture Science and Technology College, Jilin, China; ^3^Animal Husbandry and Veterinary Station in Huzhu County of Qinghai Province, Haidong, China

**Keywords:** mink, CAdV-1, 100 K protein, bioinformatics analysis, homology analysis

## Abstract

**Introduction:**

Animal trade favors the spreading of emerging canine adenovirus 1 (CAdV-1) in mink. Because the 100K protein is not exposed to the viral surface at any stage, it can be used to differentiate the vaccine from wild virus infection. However, no related research has been conducted. This study aimed to find evidence of CAdV-1 in mink and predict the character of the 100K protein in the current circulating CAdV-1 strain of mink.

**Method:**

In this experiment, the identification of CAdV-1, the phylogenetic tree, homology, and bioinformatics analysis of 100K were conducted.

**Results:**

The results showed that the CAdV-1 was identified in the mink and that its Fiber was located in a separate branch. It was closely related to strains isolated from Norwegian Arctic fox and Red fox. 100K was located in a separate branch, which had the closest genetic relationship with skunks, porcupines, raccoons, and hedgehogs and a far genetic relationship with the strains in dogs. 100K protein is an unstable and hydrophobic protein. It had evidence of selective pressure and recombination, 1 glycosylation site, 48 phosphorylation sites, 60 dominant B cell epitopes, and 9 peptides of MHC-I and MHC-II. Its subcellular localization was mainly in the endoplasmic reticulum and mitochondria. The binding sites of 100K proteins were DBP proteins and 33K proteins.

**Discussion:**

The stains in the mink were different from fox. The exploration of its genomic characteristics will provide us with a deeper understanding of the prevention of canine adenovirus.

## Introduction

Canine adenovirus type 1 (CAdV-1) can cause systemic and potentially fatal viral diseases in domestic dogs and wildlife (Mira et al., 2022). CAdV-1 can cause infectious hepatitis in dogs. Clinical symptoms include tonsillitis, vasculitis, and bladder edema (Decaro et al., 2007; Hornsey et al., 2019). Mink, as a representative animal, is widely distributed and plays a crucial role in the transmission of various viral diseases (Oude Munnink et al., 2021). However, limited data showed the surveillance and prevalence of CAdV-1 in mink.

Canine adenovirus has icosahedral symmetry and is a non-enveloped double-stranded DNA virus (32 kb) with a size of 90–100 nm (Benko et al., 2022). The 100 K protein is one of the most abundant non-structural proteins in late-stage cells after adenovirus infection and is a major component of the cell plasma. Assembly of the adenovirus capsid protein hexon depends on the assistance of the molecular chaperone L4-100 K (Shah et al., 2017). L4-100 K was involved in the hexon translation process and could prevent hexon degradation by the proteasome in co-transfected human cells. The 100 K protein transports hexa-neighbor proteins from the cytosol to the nucleus for assembly (Cepko and Sharp, 1983). If the 100 K protein is mutated or missing, the adenovirus cannot be assembled into a mature viral particle (Li et al., 1998). Therefore, it indicates that the 100 K protein promotes hexon assembly and translation and inhibits hexon degradation in FAdV-4 and HAdV-5. However, it is elusive in the CAdV-1. The 100 K protein is not exposed to the viral surface at any stage (Shah et al., 2016). Therefore, it can be used to differentiate between the vaccine and the infection. However, limited data showed the character of the 100 K protein in CAdV-1.

In this experiment, Mink CAdV-1 was found in the mink, and the 100 K protein was sequenced. The character of the 100 K protein in Mink CAdV-1 was predicted and compared with it in Fox CAdV-1 using a bioinformatics approach, which is expected to provide basic data for the prevention of the disease.

## Materials and methods

### Ethics statement

The experimental procedures outlined below were reviewed, approved, and conducted in compliance with the guidelines of the IACUC of the Jilin Agriculture Science and Technology College. The approved research protocol number is LLSC202301010.

### Identification of CAdV-1 in a mink rectal swab

The rectal swabs used in this experiment were collected from 550 1-year-old minks from 18 mink farms in Shandong, Hebei, Jilin, Liaoning, and Heilongjiang provinces in China. Each farm collected 30 samples, with both males and females randomly selected. It is worth noting that there were no obvious clinical symptoms in the minks during sampling. To extract DNA, we used TransGen Biotech's Virus DNA/RNA Purification Kit (ER201-02), following the instructions. After extraction, we stored the DNA at −20°C.

### Identification of the rectal swab as CAdV-1

To distinguish between CAdV-1 and CAdV-2, CAdV-1 identification primers were designed using SnapGene software in the E3 region: HA1 508 bp (5′CGCGCTGAACATTACTACTTGTC3′) and HA2 508 bp (3′CCTAGAGCACTTCTGTCCGCTT5′). PCR amplification was conducted on 550 anal swabs of DNA samples using HA1/HA2 508bp primers. The PCR amplification program consisted of pre-denaturation at 95°C for 1 min, denaturation at 95°C for 15 s, annealing at 59°C for 30 s, and extension at 72°C for 45 s for a total of 35 cycles. After amplification, the extension step continued at 72°C for 7 min. Subsequently, nucleic acid electrophoresis was performed on a 1% agarose gel to analyze the PCR products.

### Amplification of the Mink CAdV-1 fiber gene

Amplification using DNA from mink rectal swabs cannot obtain the fiber sequence. Finally, the gene was obtained by inoculating the chicken embryos with allantoic fluid.

### Chicken embryo allantoic fluid inoculation

We took a positive anal swab sample and mixed it with PBS in a clean 1.5-ml EP tube. After shaking and mixing, we placed it at room temperature for 1 h, filtered it into a new sterile EP tube using a 0.22 ul needle filter in an ultra-clean table to achieve a total volume of about 800 ul, and stored it overnight at −20°C. Afterward, we placed the newly arrived chicken embryos in a 37°C constant temperature incubator, flipped the eggs twice a day, and observed the eggs. Then, we discarded the dead chicken embryos until they were 9–10 days old.

We placed the chicken embryo in the biosafety cabinet, found the air chamber, and disinfected the air chamber with iodine tincture and 75% alcohol cotton. We then proceeded to make a small hole above the air chamber and placed it upwards on the egg rack. Afterward, we used a 1-ml syringe to suck 0.5 ml of filtered and sterilized virus solution and slowly injected the virus solution into the chicken embryos' chorioallantoic cavity through the hole. We then sealed the holes with melted candles and placed them back in the incubator for incubation. We flipped the eggs twice a day and observed them. We discarded chicken embryos that died within 24 h. After 72 h of inoculation, the chicken embryos were placed in a 4°C refrigerator for 4 h, and the eggshells were disinfected with alcohol. The air chamber was opened with ophthalmic forceps to collect chicken embryo allantoic fluid and extract DNA.

### Construction of the mink CAdV-1 fiber and the 100 K plasmid

SnapGene software was used to design primers Fiber 956bp F (5′CCGGGTCTCACCGTACTAAACG′3), Fiber 956bp R (3′GCAGCGCCAGCTATTTGTT 5′), CAdV-1 100 K primers F (5′ATGTCAGAAGAGCCCGTCAGTG3′), and R (3′CTAGGAGGTCGTTCCTCTGACATCT5′). We used two pairs of primers to amplify chicken embryos' allantoic fluid DNA. Nucleic acid electrophoresis was performed on a 1% agarose gel. We successfully amplified the CAdV-1 Fiber and CAdV-1 100 K genes and connected them to the pMD^TM^18-T vector.

### Phylogenetic and homology analysis of the 100 K protein

The phylogenetic tree and homology analysis of the 100 K protein in the Mink and Fox CAdV-1 were conducted by MEGA 7.0 (Mega Limited, Auckland, New Zealand) and DNAstar software (DNASTAR, Madison).

### Selective pressure and recombination analysis of the 100 K protein

The GARD and SLAC programs of the DataMonkey (http://www.datamonkey.org/) online software were used to predict the selective pressure and recombination analysis in the 100 K protein in the Mink and Fox CAdV-1.

### Physical and chemical properties of 100 K protein

The physicochemical properties of the 100 K protein in the Mink and Fox CAdV-1 were predicted using the online software ProtParam (https://web.expasy.org/protparam/). The amino acids of the 100 K protein were counted separately and plotted using Origin software (OriginLab, Amherst).

### Hydrophilicity and Hydrophobicity prediction of 100 K protein

The online software ProtScale (https://web.expasy.org/prot-scale/) was used to predict the hydrophilicity and hydrophobicity sites of the 100 K protein in the Mink and Fox CAdV-1.

### 100 K protein phosphorylation site prediction

The online software Netphos-3.1 (https://services.healthtech.dtu.dk/services/NetPhos-3.1/) was used to predict the phosphorylation sites of the 100 K protein in the Mink and Fox CAdV-1.

### 100 K protein glycosylation site prediction

The online software NetNGlyc-1.0 (https://services.healthtech.dtu.dk/services/NetNGlyc-1.0/) was used to predict the glycosylation sites of the 100 K protein in the Mink and Fox CAdV-1.

### Prediction of MHC-1 and MHC-2-binding sites in 100 K protein

The MHC-I and MHC-II antigen sites of the 100 K protein in Mink and Fox CAdV-1 were predicted using the online software IEDB (https://www.iedb.org/).

### B-cell epitope prediction

The internal epitopes of 100 K in Mink and Fox CAdV-1 were predicted using IEDB (https://www.iedb.org/). The B-cell epitopes of the 100 K protein in Mink and Fox CAdV-1 were predicted with ABCpred (http://crdd.osdd.net/raghava/abcpred/). The higher the score of the peptide, the higher the probability of it being an epitope.

### Prediction of linear and non-linear epitopes of 100 K protein

The PDB file of the 100 K protein was obtained from Robetta (http://robetta.bakerlab.org/queue.jsp). The linear and non-linear epitopes of the 100 K protein were predicted using ElliPro software (https://www.iedb.org/). The higher the score, the higher the solvent-accessible surface area of the residue.

### 3D docking of the 100 K protein

The upstream protein DBP and downstream protein 33K of the 100 K protein in CAdV-1 were obtained from Robetta (http://robetta.bakerlab.org/queue.jsp). 3D simulation docking of DBP, 100 K, and 33K were performed using PYMOL software (DeLano Scientific LLC, NewYork).

### Nuclear localization of the 100 K protein

Nuclear localization was predicted using the online software Genscript (https://www.genscript.com/psort.html).

## Results

### Identification of CAdV-1 in a mink rectal swab

**Figure 1:**
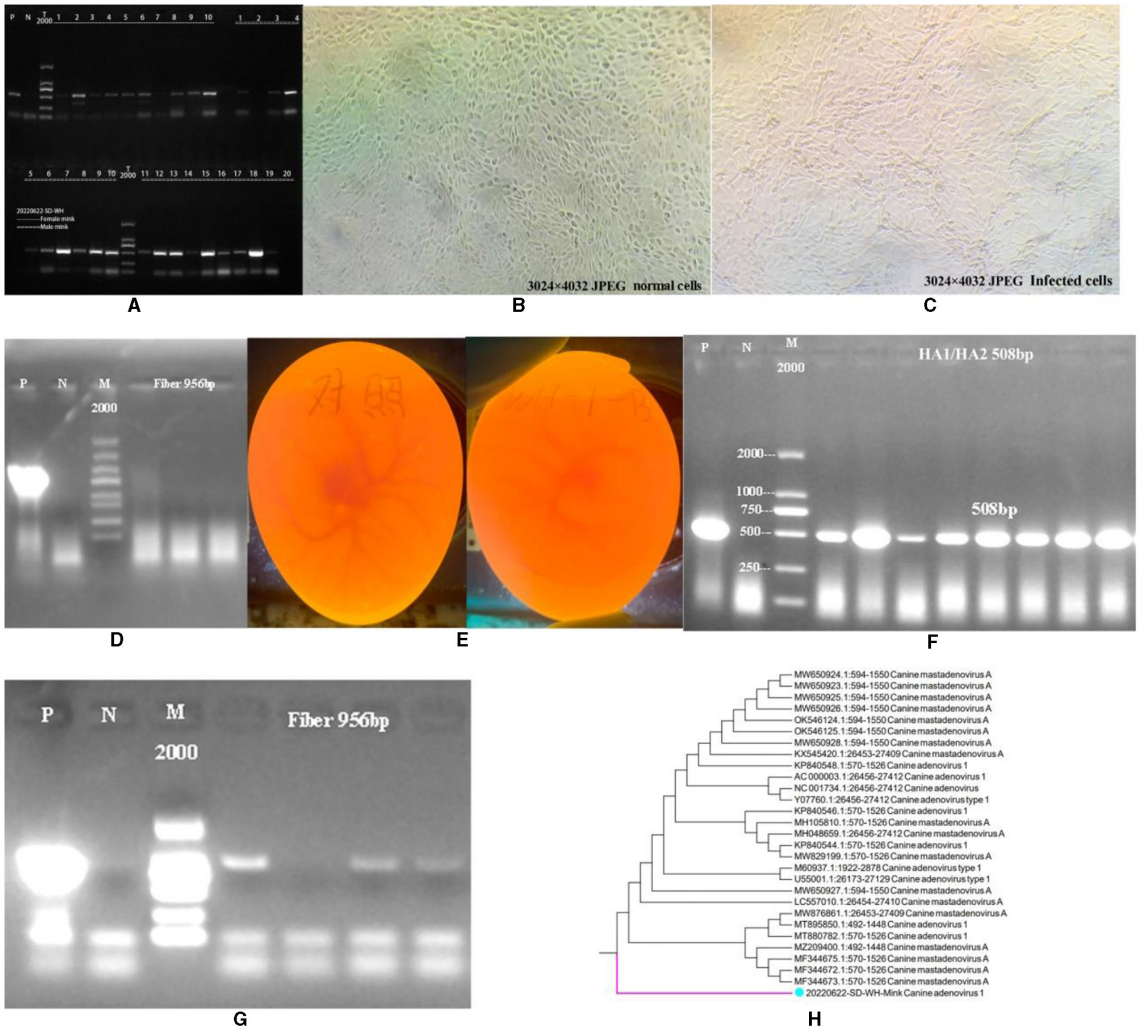
Identification of CAdV-1. **(A)** Identification of CAdV-1 in mink (P is a positive control, and N is a primer control.). **(B)** Normal MDCK. **(C)** MDCK after being poisoned. **(D)** Identification of CAdV-1 in cell supernatant (From left to right, there are the first, second, and third doses of poisoning, P is a positive control; N is a primer control, the amplification length is 956 bp.). **(E)** On the left is the control group's chicken embryo. On the right is the chicken embryo 72 h after inoculation with the premix of mink rectal swabs from Weihai City, Shandong Province, China. **(F)** Identification of CAdV-1 in chicken embryo allantoic fluid DNA (P is a positive control, and N is a primer control, the amplification length is 508 bp.). **(G)** Identification of CAdV-1 Fiber in Chicken Embryos Allantoic Fluid DNA (P is a positive control, and N is a primer control, the amplification length is 956 bp.). **(H)** Phylogenetic Tree of Mink CAdV-1 Fiber.

As shown in Figure 1A, CAdV-1 was detected in 30 minks from a mink farm in Weihai City, Shandong Province, China. The results showed that 23 minks were positive for CAdV-1, with a positive rate of 76.67%. We attempted to isolate the mink CAdV-1 Fiber gene by inoculating MDCK. As shown in Figures 1B, C, the cells did not produce obvious lesions. As shown in Figure 1D, the cell supernatant was extracted for CAdV-1 identification, but CAdV-1 was not detected. Figure 1E is the inoculation map of the chicken embryo, and Figure 1F is the identification result of Mink CAdV-1 in chicken embryo allantoic fluid. The results showed that Mink CAdV-1 was successfully cultured in the chicken embryo. Figure 1G is the identification result of the Mink CAdV-1 Fiber gene in chicken embryo allantoic fluid, which shows that the Mink CAdV-1 Fiber gene was successfully detected.

### Construction of the mink CAdV-1 fiber phylogenetic tree

Based on the obtained sequences and other sequences in the NCBI database, a phylogenetic tree was constructed, and it was found that the Mink CAdV-1 Fiber obtained in this experiment was located in a separate branch. Figure 1H is the phylogenetic tree of the Mink CAdV-1 Fiber gene. The results showed that it was located in a separate branch and had the closest genetic relationship with wild animals such as foreign foxes. It is closely related to MF344673.1, MF344672.1, and MF344675.1 strains isolated from the Norwegian Arctic and red foxes. Moreover, it is closer to the Melurus ursinus strain MZ209400.1 from India, the Canis lupus familiaris strains MT880782.1 and MT895850.1 from India, and the Jackal strain MW876861.1 from India. The farthest phylogenetic relationships with Italian dog isolates MW650924.1, MW650923.1, and MW650928.1 were observed, followed by Canis lupus isolates OK546124.1 and OK546125.1 from the Northwest Territories of Canada.

### Construction of the 100 K plasmid

**Figure 2:**
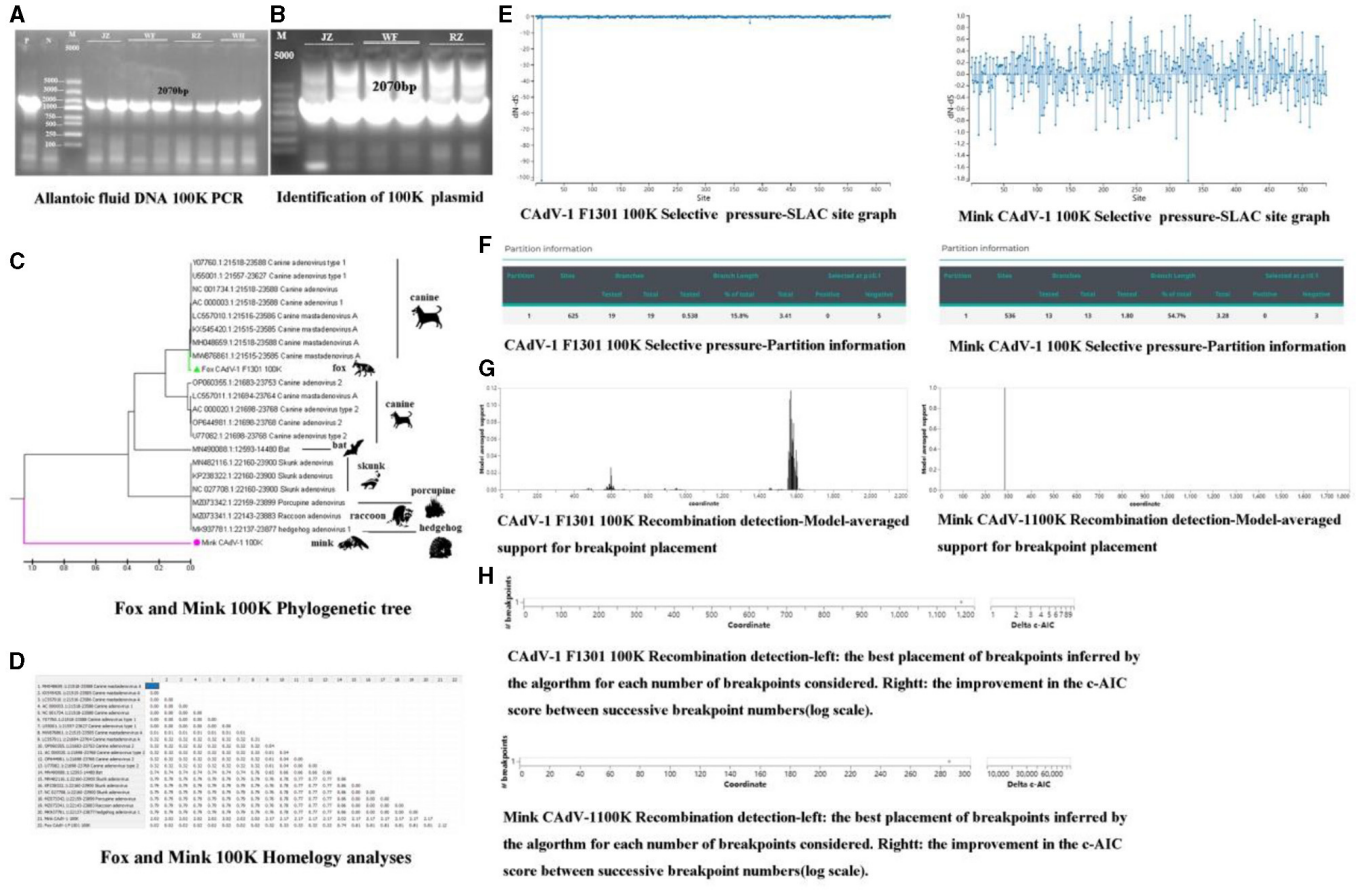
The phylogenetic analysis, selective pressure, and recombination of the 100 K protein. **(A)** The amplification of 100 K (P is a positive control, and N is a primer control. The amplification length is 2070 bp. JZ and WF represent Jinzhou City and Wafang City, Liaoning Province, and RZ and WH represent Rizhao City and Weihai City, Shandong Province.). **(B)** The identification of 100 K plasmids (P is a positive control, and N is a primer control. The amplification length is 2070 bp. JZ and WF represent Jinzhou City and Wafang City, Liaoning Province, and RZ represent Rizhao City, Shandong Province.). **(C)** The Phylogenetic tree of 100 K in Mink and Fox CAdV-1. **(D)** Homology analysis of 100 K in Mink and Fox CAdV-1. **(E)** Selective pressure-SLAC site graph. **(F)** Selective pressure-partition information in Mink and Fox CAdV-1. **(G)** Recombination detection-model-averaged support for breakpoint placement in Mink and Fox CAdV-1. **(H)** Recombination detection in Mink and Fox CAdV-1.

The gel imaging system showed a bright band of 100 K (Figure 2A). It was inserted into the 18T vector, and the 100 K protein was amplified in the 18T plasmid. It indicated that the mink CAdV-1 100 K plasmid was successfully constructed (OQ981364) (Figure 2B).

### Phylogenetic and homology analyses of the 100 K protein

As shown in Figures 2C, D, the fox CAdV-1 100 K protein was located in the same branch as the CAdV-1 in dogs, and the genetic distance was 0.00, which might be a paralogous gene. Mink CAdV-1 100 K protein was located in a separate branch, which had the closest genetic relationship with Skunks, Porcupines, Raccoons, and hedgehogs and had a far greater genetic relationship with CAdV-1.

### Selective pressure and recombination analysis of the 100 K protein

There were 666 potential breakpoints, and the model search space of 2 breakpoints was further searched. The best breakpoint position was between 1,150 and 1,200. Mink CAdV-1 100 K analysis showed that there were 1,824 potential breakpoints, the model search space of two breakpoints was further searched, and the optimal breakpoint position was between 280 and 300 (Figures 2E, F). There were two breakpoint placements at sites 600 and 1,600 in the recombination analysis of Fox CAdV-1. There was one breakpoint placement at site 300 in the recombination analysis of Mink CAdV-1 (Figures 2G, H).

### Physical and chemical properties of 100 K protein

**Figure 3:**
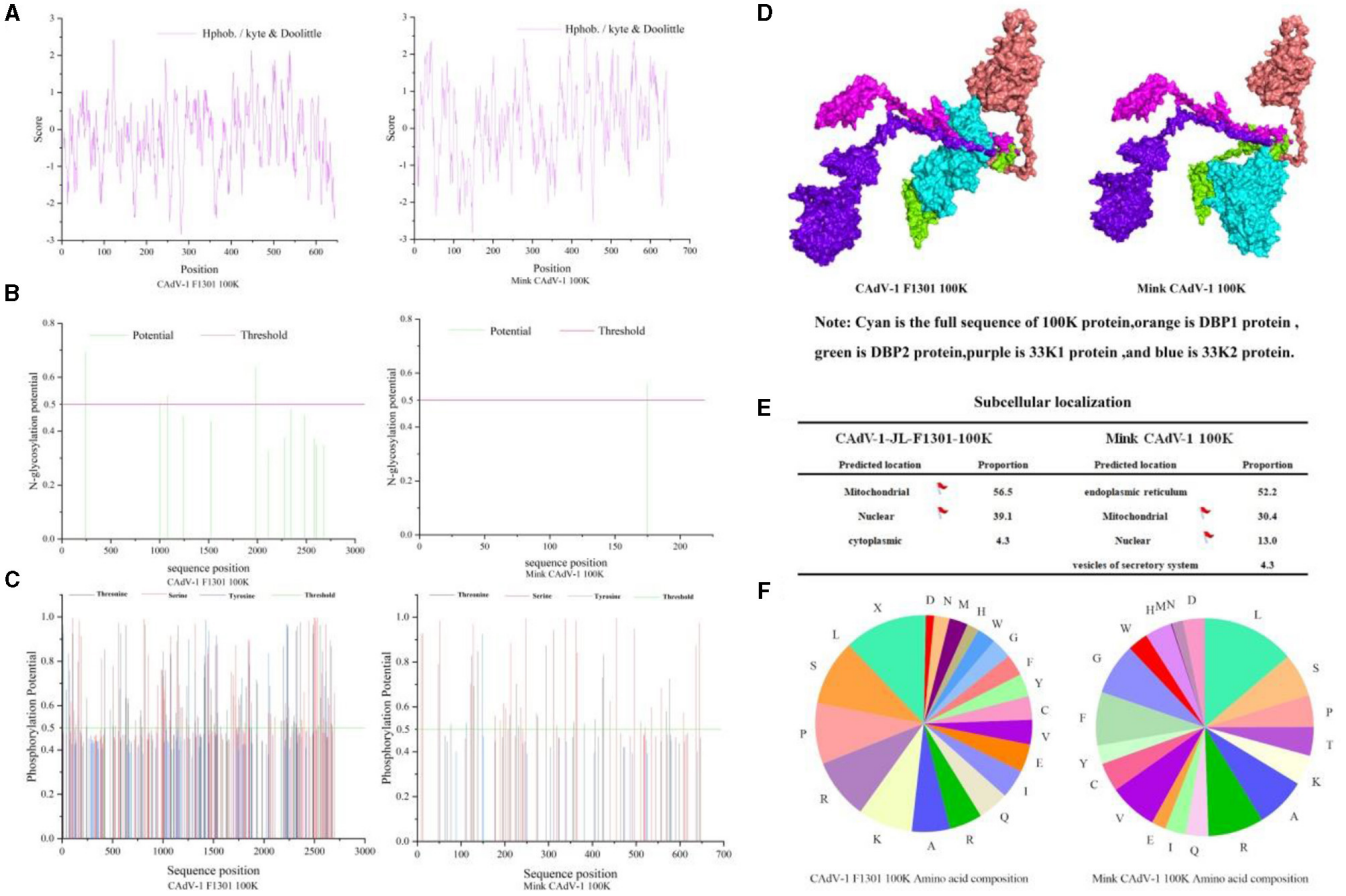
Character of the 100 K protein in Mink and Fox CAdV-1. **(A)** Hydrophobicity prediction of 100 K protein. **(B)** Glycosylation site prediction of the 100 K protein. **(C)** Phosphorylation site prediction of the 100 K protein. **(D)** 3D docking of the 100 K protein. **(E)** Nuclear localization of the 100 K protein. **(F)** The proportion of amino acids in the 100 K protein.

The molecular formula of the Mink CAdV-1 100 K protein was C_3436_H_5255_N_949_O_845_S_29_, the relative molecular mass was 74308.11, the isoelectric point was 9.93, the total number of amino acid residues with positive and negative charges was 83 and 35, respectively, and the instability index was 41.85. The 100 K protein in Fox CAdV-1 is similar to that of Mink CAdV-1 in the molecular formula, relative molecular mass, isoelectric point, and positively and negatively charged amino acid residues. The 100 K protein in mink CAdV-1 is unstable, but in fox CAdV-1, it is not. Proline was the highest, and leucine was the lowest in the fox 100 K protein. The proportion of leucine in mink 100 K protein was the highest, and the proportion of methionine was the lowest in mink CAdV-1 (Figure 3F).

### Hydrophilicity and Hydrophobicity prediction of 100 K protein

As shown in Fig.3 A, the average hydrophilic coefficient of the Fox CAdV-1 100 K protein was−0.254, indicating that the protein was hydrophilic. The average hydrophilicity coefficient of the Mink CAdV-1 100 K protein was 0.143, indicating that the protein was hydrophobic. The 100 K protein was different between Mink and Fox CAdV-1.

### 100 K protein glycosylation site prediction

As shown in Figure 3B, the Fox CAdV-1 100 K protein had five glycosylation sites located at positions 246, 1,981, 2,110, 2,281, and 2,678, while the Mink CAdV-1 100 K protein had only one glycosylation site located at position 175.

### 100 K protein phosphorylation site prediction

As shown in Figure 3C, Fox CAdV-1 100 K protein had 53 serine phosphorylation sites, 43 threonine phosphorylation sites, and 6 tyrosine phosphorylation sites, for a total of 102. The Mink CAdV-1 10 0K protein had 28 serine phosphorylation sites, 18 threonine phosphorylation sites, and 2 tyrosine phosphorylation sites, for a total of 48.

### 3D docking of the 100 K protein

As shown in Figure 3D, it was found that the binding sites of Fox 100 K protein and mink 100 K protein were the same as those of DBP protein and 33K protein.

### Subcellular localization of the 100 K protein

The subcellular localization of Fox and Mink CAdV-1 100 K proteins was mainly in the endoplasmic reticulum and mitochondria (Figure 3E).

### Prediction of MHC-1-binding and MHC-2-binding in the 100 K protein

**Figure 4:**
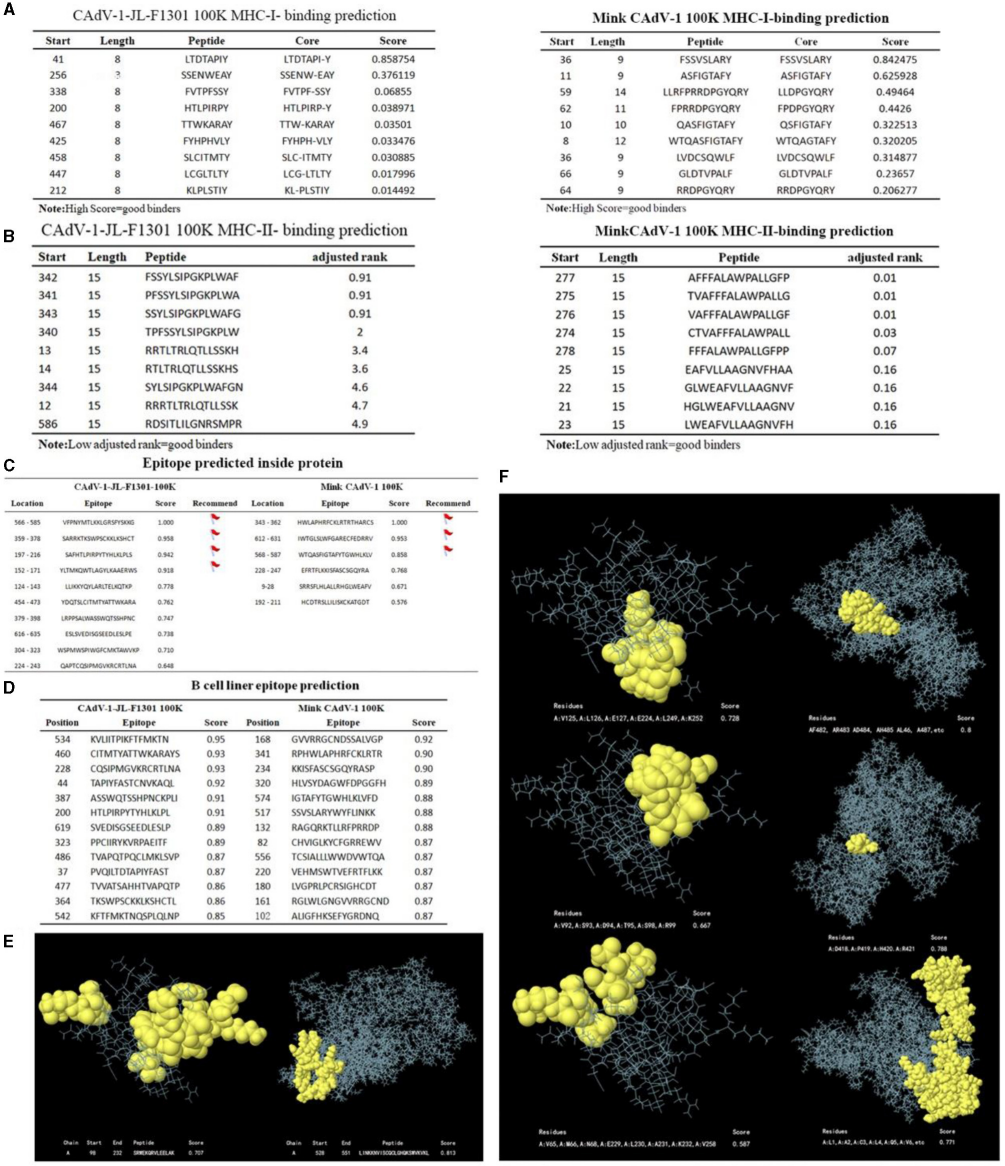
The prediction of MHC-I and MHC-II, the epitopes of the 100 K protein. **(A)** MHC-I-binding prediction. **(B)** MHC-II-binding prediction. **(C)** Epitope prediction inside the protein. **(D)** B-cell linear epitope prediction. **(E)** Linear structure of 100 K. The linear structure of the CAdV-1 F1301 100 K protein is on the left. The linear structure of the Mink CAdV-1 100 K protein was on the right. **(F)** The non-linear structure of 100 K. The non-linear structure of the CAdV-1 F1301 100 K protein is on the left. The non-linear structure of the Mink CAdV-1 100 K protein was on the right. Moreover, the score from top to bottom was from high to low.

Mink CAdV-1 100 K protein and Fox CAdV-1 100 K protein had nine peptides of MHC-I and MHC-II. However, the affinity of MHC-I and MHC-II for the Mink CAdV-1 100 K protein was significantly better than that of the Fox CAdV-1 100 K protein. The specific scores are shown in Figures 4A, B.

### B-cell epitope prediction

The table marked the epitopes inside the protein with a score of more than 0.8 points. To accurately synthesize the antigen, the above sites should be avoided as much as possible (Figure 4C). All peptides shown here were above the selected threshold (0.5). The Fox CAdV-1 100 K protein showed 66 dominant B-cell epitopes. Mink CAdV-1 100 K had 60 dominant B-cell epitopes (Figure 4D).

### Prediction of linear and non-linear epitopes of 100 K protein

As shown in Figures 4E, F, continuous and discontinuous epitopes of the 100 K protein were predicted from the 3D structure of the 100 K protein. The linear epitopes with the first score and the non-linear epitopes with the first three scores.

## Discussion

Canine adenovirus type 1 (CAdV-1) is the causative agent of systemic and potentially fatal viral disease in mink. The co-infection with canine distemper virus (CDV) and canine adenovirus type 1 (CAdV-1) was the first description of a free-living hoary fox pup from Brazil (Silva et al., 2023). The genetic heterogeneity of CAdV-1 was found in Italy (Mira et al., 2022).

CAdV-1 was first discovered in mink rectal swabs. In previous experiments, to obtain the fiber sequence, the sample was inoculated with MDCK, the MDCK was blindly cultivated for five generations, and their supernatants were identified. The presence of CAdV-1 was not found. Interestingly, the fiber gene was successfully amplified by inoculation with chicken embryos' allantoic fluid. It was found that the mink CAdV-1 Fiber obtained in this experiment was located in a separate branch. It is closely related to strains isolated from Norwegian and red Arctic foxes. It indicates that the CAdV-1 in the mink is transferred from the fox. Although the CAdV-1 Fiber sequence of mink is similar to that of fox, which belongs to canines, it is not cultivated on MDCK. Mink belongs to a weasel, and it is speculated that there is still a species issue, resulting in mink CAdV-1 not proliferating on MDCK. This also leads to the possibility that there is no corresponding receptor for mink CAdV-1 in MDCK.

After adenovirus infection, the 100 K protein was one of the most abundant non-structural proteins in late-stage cells. Moreover, 84–133 and 656–697 were two non-adjacent domains on the N-terminus and C-terminus of human adenovirus type 5 L4-100 K, which were crucial for the expression and assembly of hexon trimers (Zhu et al., 2023). The human adenovirus type 5 (HAdV-5) late-stage L4-100 K protein has a regulatory reversible acetylation mechanism (Xu, 2022). The HAdV-5 100 K protein specifically forms a stable complex with granzyme B, inhibits apoptosis in infected cells, and promotes viral replication (Andrade et al., 2001). The interaction of the avian adenovirus 100 K protein with the HSC70 protein promotes the replication of avian adenovirus serotype 4 in LMH cells (Gao et al., 2022). Therefore, the 100 K protein promotes the assembly, translation process, transportation, trimer expression, and reversible acetylation of the Hexon. The 100 K protein inhibits cell apoptosis and promotes viral replication and assembly. It indicates that the 100 K protein plays a critical role in adenovirus infection. The 100 K protein is not exposed to the viral surface. It can be used to differentiate between a vaccine and a wild virus infection.

100 K, as an important scaffold protein, can trimer the main capsid Hexon of the virus and promote the generation of progeny virion in target cells (Ackford et al., 2017). Although the 100 K protein plays an important role in CAdV-1, there has been no systematic research on it to date. Bioinformatics is a tool discipline used to analyze biological data, specifically to analyze and integrate markers. In this experiment, the 100 K sequence was obtained, and its character was predicted using bioinformatics. According to the phylogenetic tree, Fox CAdV-1 is related to the canine adenovirus. Mink CAdV-1 100 K protein was located in a separate branch from Fox CAdV-1 100 K protein, which had the closest genetic relationship with hedgehogs, raccoons, porcupines, and skunks and had a far more distant genetic relationship with dogs. As the 100 K protein was the conserved sequence, it indicated that the Mink CAdV-1 was transmitted from hedgehogs, raccoons, porcupines, and skunks. According to the homology analyses, the distance between Mink CAdV-1 and other CAdV-1 strains was far. It indicated that Mink CAdV-1 was a different novel strain. The proportion of leucine in Mink 100 K protein was the highest, and the proportion of methionine was the lowest. It is different from the Fox 100 K protein. High concentrations of leucine can inhibit the NF-κb pathway and phosphorylation level, inhibit virus replication, and promote cell proliferation (Wang, 2019). It contributes to the difference between Fox and Mink CAdV-1 100 K.

Mutation and recombination, as the main molecular forces driving the adaptation of viruses to their environment, have become hot topics in the study of viral evolution. The maximum likelihood method test for detecting selection pressure has been widely accepted, where the selection coefficient, ω (ω denotes the non-synonymous/synonymous substitution rate ratio, dN/dS), visually reflects the codons ω > 1, ω = 1 and ω < 1, which represent the evolutionary trend of the organism at positive selection, neutral selection, and negative selection (purifying selection) during the evolutionary process. Positive selection by host-specific immune pressure contributes to the mutation of viral proteins, thus facilitating the viral evasion of host immune recognition and adaptive purification (Yang and Bielawski, 2000). Therefore, understanding the evolutionary process of viral genetic diversity is essential to improving the effectiveness of vaccine control. Mink CAdV-1 100 K analysis showed that there were 1,824 potential breakpoints, the model search space of two breakpoints was further searched, and the optimal breakpoint position was between 280 and 300. Many studies have now shown that positive selection from host-specific immunity pressure helps viral proteins mutate and thus allows viruses to evade host immune recognition and adaptive evolution (Suzuki, 2006). These selection sites detected on the 100 K proteins of CAdV-1 could be evidence for the adaptive evolution of CAdV-1 as a target for viral evasion of the host immune response. The analysis of these selection sites could have a positive effect on the identification of immune evasion, drug resistance mutations, and the prevention of viral diseases.

Recombination regulates the evolutionary process of the virus. GARD is an extensible and intuitive method to screen sequences for recombination. In this experiment, there was one breakpoint placed at site 300 in the recombination analysis of Mink CAdV-1. Genetic recombination is widespread in viral evolution and is an important driver of viral genetic variation. New viruses resulting from genetic recombination will be more conducive to viruses acquiring greater pathogenicity, possibly in combination with the original viral disease pandemics due to compound infestation with the original virus (Saunders et al., 2000). Recombination sites may lead to changes in antigenic epitopes, a way for viruses to mutate and create new populations, and a cause of immune failure (Vlasova et al., 2014). This situation may lead to ineffective or failed immunization with existing CAdV-1 vaccines or the failure of existing CAdV-1 vaccines and, more likely, the emergence of novel mutant strains. Genetic recombination may lead to changes in the antigenic epitopes of viruses that lead to virus mutation, which is both a way for viruses to mutate to create new subgroups and an important cause of vaccine-specific immunization failure. Therefore, CAdV-1 sampling and recombination analysis are essential to preventing possible future CAdV-1 outbreaks.

Hydrophobic and hydrophilic are the basic characteristics of the protein. Hydrophobic residues preferentially occur within proteins, while hydrophilic residues tend to occur on protein surfaces. They are the main driving forces of protein folding and affect structure and function (Tang et al., 2021). The hydrophilicity distribution of proteins can reflect the folding of the protein. In the overall folding structure of the protein, a highly hydrophobic region is formed in the transmembrane region. Based on this, the position of the secondary structure, such as the transmembrane helix of the protein, can be determined. In this experiment, the 100 K protein was found to be a hydrophilic protein, and the hydrocolloid property of the 100 K protein can be used to purify the 100 K protein by dialysis. The 100 K protein can be separated and purified by electrophoresis according to the obtained isoelectric point (Zheng et al., 2020). Hydrophobic residues are often present inside the protein, and hydrophilic residues are often present on the surface of the protein. The protein antigen epitope is closely related to the protein hydrophilic site, but the high hydrophilic site is not necessarily related to the epitope (Matsui et al., 2017). On the protein surface, the actual distribution of hydrophobic residues modifies the molecular structure of the protein to eliminate the restriction of spatial distribution and expose more hydrophobic residues on the molecular surface. The spatial structure controls the surface hydrophobicity of proteins. The change in surface hydrophobicity caused by the change in molecular structure can be used as an ideal key indicator to predict and evaluate the change in protein surface properties (Tang et al., 2021). Although this parameter is not very important in epitope prediction, when using synthetic peptides to prepare antibodies, the hydrophobicity is too strong, which may make it difficult to synthesize the correct peptides. Thus, hydrophobic sites can be avoided to synthesize the correct peptides.

Protein phosphorylation is a common protein modification method that can convert exogenous stimuli into endogenous signals, help open potassium channels, and participate in enzymatic reactions. Protein phosphorylation plays an important role in cell cycle regulation, signal transduction, and metabolic pathways (Bilbrough et al., 2022). Phosphorylation of Ser, Thr, and Tyr constitutes O-phosphorylation, which is very stable under acidic conditions and therefore has been widely studied in cell biology and phosphorylated proteomics (Bilbrough et al., 2022). In this experiment, the Mink CAdV-1 100 K protein had 48 phosphorylation sites. Moreover, 48 phosphorylation sites of Mink CAdV-1 were lower than those of 100 K in Fox CAdV-1. It indicates that Mink CAdV-1 100 K is different from that in Mox CAdV-1. The 100 K protein, a specific adenovirus protein with 48 phosphorylation sites, may be associated with intracellular signal transduction and protein localization.

Glycosylation is a posttranslational modification responsible for cell cycle regulation, protein–protein interactions, and cell signaling (Benešová et al., 2022). Glycosylation is also an important modifying effect of proteins, which plays a role in regulating protein function and thus inducing a high level of natural immune response (Eichler, 2019). The development of lectin microarray technology provides convenience for the study of the structure and function of glycans and glycosylation. Studies have shown that the newly developed equipment can perform high-speed and accurate research on abnormal glycosylation (Bangarh et al., 2023). Although glycosylation accounts for 2–3% of the total mass of IgG antibodies, its therapeutic application is mainly attributed to the N-glycosylation profile of a monoclonal antibody (mAb) (Mota et al., 2022). The Mink CAdV-1 100 K protein has only one glycosylation site. Because there are no signal peptides, the protein is not exposed to the N-glycosylation mechanism. Although this sequence has potential glycosylation sites, it may not be glycosylated *in vivo* because it does not contain a signal peptide.

At the base of N-linked and O-linked glycosylation, bacteria and viruses exert critical biological functions. Glycosylation of viral proteins has multiple functions. N-linked glycosylation is considered to be the most common way of protein modification, playing a key role in regulating protein folding, transport, and receptor binding. However, the function of O-linked viral protein glycosylation has not been fully reported (Feng et al., 2022). Studies have reported that oligosaccharides can covalently bind to viral envelope proteins, which is crucial in determining host–virus interactions. However, it is not yet clear how glycans utilize host immune mechanisms and assist receptors in mediating viral invasion (Routhu and Byrareddy, 2017; Routhu et al., 2019). Thus, the only glycosylation site is also vital for protein folding, transportation, and receptor binding in the mink CAdV-1.

B cells are mainly involved in humoral immunity. Both B-cell epitopes and antigenic determinants could provide a basic theory for the development of a canine adenovirus 100 K protein-based antigenic epitope vaccine. MHC-I molecules are distributed on the surface of all cells containing the nucleus, and MHC-II is distributed on the surface of the antigen, both of which are mainly responsible for antigen presentation. MHC-II displays peptides to T helper cells on the surface of professional antigen-presenting cells. Identifying T-cell epitopes through the peptides presented by the MHC-II molecule (Jensen et al., 2018) is important. The possible B-cell epitopes of candidate molecules are predicted theoretically using computer-aided molecular design technology. The results showed that the affinity of MHC-I and MHC-II for Mink CAdV-1 100 K protein was significantly better than that of Fox CAdV-1 100 K protein. Jia identified CD2v B-cell epitopes using scanning peptides and bioinformatics (Jia et al., 2022). By predicting and calculating the potential B and T cell epitopes of several EBV proteins, these proteins could mediate epithelial cell attachment and diffusion, capsid self-assembly, DNA synthesis, and replication (Olotu and Soliman, 2021). This greatly shortens the cycle of screening antigen epitopes and provides a new approach for the reasonable design and development of pathogen vaccines.

B-cell epitopes and antigenic determinants can provide a basic theory for the development of epitope vaccines based on the canine adenovirus 100 K protein. Antigenic determinant clusters are structures that produce immunogenicity in proteins. In this experiment, the 21 antigenic determinant clusters can interact with antibodies and activate T-cell immune responses. The 60 dominant B-cell antigenic epitopes can promote the binding of the 100 K protein to other proteins, enhancing the body's cellular and humoral immunity. Mink CAdV-1 100 K has 60 dominant B-cell epitopes. It is the predicted 3D structure mapping of continuous and discontinuous epitopes of the 100 K protein. We selected the linear epitopes with the first score and the non-linear epitopes with the first three scores. The bioinformatics analysis of the 100 K protein in this experiment is only a preliminary prediction based on existing databases, and further experiments are needed to verify the reliability of the prediction.

Molecular docking can be understood as simulating the real process of random motion of two molecules in an aqueous solution, which is helpful in understanding the interaction between molecules and predicting the binding position of molecules. It is a process in which molecules with three-dimensional structures can be inserted into the site of the receptor molecule. By continuously optimizing the position and conformation of the receptor compound, adjusting the dihedral angle of the intramolecular rotatable bond, and modifying the side chain and skeleton of the amino acid residue in the receptor, researchers take crucial steps in selecting the best conformation of receptors and ligands and predicting their binding mode and affinity. The ligand with the highest affinity for the receptor is selected using a scoring function that approximates the natural conformation. In this experiment, it was found that the binding sites of the mink 100 K protein were the same as those of the DBP protein and the 33K protein. Proteins are synthesized in the cytoplasm, but some proteins need to be localized to the nucleus to function, which is determined by the nuclear localization signal (NLS). NLS can be located at any position of the protein, generally on the branch chain of the protein; that is, it needs to be exposed and recognized to achieve nuclear translocation. Gene editing can be achieved by adding or missing NLS to achieve nuclear translocation expression of endogenous and exogenous proteins. In this experiment, the NLS of Fox CAdV-1 100 K protein was mainly in mitochondria, and its proportion in the nucleus was much higher than that of Mink CAdV-1 100 K. The NLS of Mink's CAdV-1 100 K protein was mainly in the endoplasmic reticulum and mitochondria. This result proves that the 100 K protein has nuclear import ability, which provides important information for studying the function of the 100 K protein in CAdV-1 replication.

## Conclusion

Mink CAdV-1 was first identified, and it was on a separate branch of the phylogenetic tree. The 100 K protein was located in a separate branch, which had the closest genetic relationship with skunks, porcupines, raccoons, and hedgehogs and had a far greater genetic relationship with CAdV-1. The mink CAdV-1 100 K protein had evidence of select pressure and recombination. The 100 K protein in Mink CAdV-1 is an unstable and hydrophobic protein. The proportion of leucine in mink 100 K protein was the highest, and the proportion of methionine was the lowest in mink CAdV-1. The Mink CAdV-1 100 K protein had only one glycosylation site and 48 phosphorylation sites. The binding sites of the mink 100 K protein were the DBP protein and the 33K protein. The subcellular localization of a Mink CAdV-1 100 K protein was mainly in the endoplasmic reticulum and mitochondria. Mink CAdV-1 100 K had 60 dominant B-cell epitopes. Mink CAdV-1 100 K protein had nine peptides of MHC-I and MHC-II. The continuous and discontinuous epitopes of the 100 K protein were predicted from the 3D structure of the 100 K protein. Mink CAdV-1 100 K was different from that in fox CAdV-1. Our study provides preliminary structural and functional predictions for the biological effects of the 100 K protein, and further research to develop a 100 K subunit vaccine is essential to differentiate the vaccine from infection.

## Data availability statement

Original datasets are available in a publicly accessible repository: The original contributions presented in the study are publicly available. This data can be found here: BankIt2703237 Seq1 OQ981364 in the NCBI repository.

## Ethics statement

The animal study was reviewed and approved by IACUC of the Jilin Agriculture Science and Technology College.

## Author contributions

All authors listed have made a substantial, direct, and intellectual contribution to the work and approved it for publication.
